# Hierarchical self-assembly of Au-nanoparticles into filaments: evolution and break

**DOI:** 10.1039/d4ra04100c

**Published:** 2024-08-28

**Authors:** Matteo Tiberi, Francesca Baletto

**Affiliations:** a Physics Department, King’s College London Strand WC2R 2LS UK; b Cambridge Graphene Centre, University of Cambridge Cambridge UK; c Physics Department, University of Milan 20133 Italy francesca.baletto@unimi.it

## Abstract

We compare the assembly of individual Au nanoparticles in a vacuum and between two Au(111) surfaces *via* classical molecular dynamics on a timescale of 100 ns. In a vacuum, the assembly of three nanoparticles used as seeds, initially showing decahedral, truncated octahedral and icosahedral shapes with a diameter of 1.5–1.7 nm, evolves into a spherical object with about 10–12 layers and a gyration radius ∼2.5–2.8 nm. In a vacuum, 42% show just one 5-fold symmetry axis, 33% adopt a defected icosahedral arrangement, and 25% lose all 5-fold symmetry and display a face-centred-cubic shape with several parallel stacking faults. We model a constrained version of the same assembly that takes place between two Au(111) surfaces. During the dynamics, the two Au(111) surfaces are kept fixed at distances of 55 Å, 55.5 Å, 56 Å, and 56.5 Å. The latter distance accommodates 24 Au layers with no strain, while the others correspond to nominal strains of 1.5%, 2.4%, and 3.3%, respectively. In the constrained assembly, each individual seed tends to reorganize into a layered configuration, but the filament may break. The probability of breaking the assembled nanofilament depends on the individual morphology of the seeds. It is more likely to break at the decahedron/icosahedron interface, whilst it is more likely to layer with respect to the (111) orientation when a truncated octahedron sits between the decahedron and the icosahedron. We further observe that nanofilaments between surfaces at 56 Å have a >90% probability of breaking, which decreases to 8% when the surfaces are 55 Å apart. We attribute the dramatic change in probability of breaking to the peculiar decahedron/icosahedron interface and the higher average atomic strain in the nanofilaments. This *in silico* experiment can shed light on the understanding and control of the formation of metallic nanowires and nanoparticle-assembled networks, which find applications in next-generation electronic devices, such as resistive random access memories and neuromorphic devices.

## Abstract 2

We compare the assembly of individual Au nanoparticles in a vacuum and between two Au(111) surfaces *via* classical molecular dynamics on a timescale of 100 ns.

## Graphical abstract

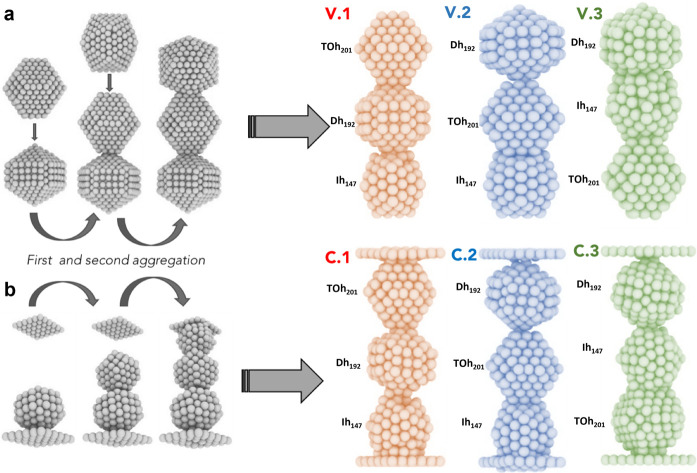

## 1 Introduction

Artificial intelligence (AI) applications require novel hardware platforms with brain-like connectivity patterns to overcome the limitations of von Neumann computing hardware.^1,2^ Non-volatile memories, such as resistive random-access memories (ReRAMs), are a key element of neuro-inspired computing. ReRAMs store information by switching between high- and low-resistance states. Resistive switching can occur *via* different mechanisms depending on the materials used and device architecture,^3^ hence ReRAMs may operate in fundamentally different ways. Whilst they are often formed *via* a metal–insulator–metal structure between two electrodes,^3,4^ devices employing phase-change materials,^5^ 2D layered materials,^6–9^ nanoassembled metallic nanoparticles^10–16^ and metallic nanowire networks^17,18^ have been demonstrated. Metallic networks reduce device fabrication complexity, as they can be prepared in solution^18,19^ and then coated on a target substrate with electrodes, or *via* gas-phase deposition directly on the target electrodes.^13,16,20^ In contrast with other ReRAM architectures, metallic ReRAMs are made of one material only, with the electrodes and active area having different electronic properties. To develop metallic network-based devices, it is critical to control the assembly of individual nanoparticles into nanofilaments. Such opportunities cause new excitement in Au-nanoparticle research. Nanoparticle self-assembly has been addressed as a new material development tool, as recently reviewed by ref. 21 and 22. Nanoparticles show extraordinary morphological diversity, depending on their formation process. Further, they can be assembled into a 1D array known as a nanofilament,^17,23–26^ or into a 3D array, namely a nanofoam.^23,27,28^ To explain, from an atomistic point of view, the properties of nanofilaments for ReRAM devices and to reveal the origin of the switching, transmission electron microscopy (TEM) and atomic force microscopy (AFM) are helpful tools to monitor the formation and diffusion of vacancies and interfaces, and even the spontaneous breaking of metallic filaments.^15,26^ TEM, as well as optical spectroscopy, have identified uniaxial assembly of nanoparticles.^29,30^ Nonetheless, a proper atomistic description shedding light on the relationship between nanofilament breaking and resistive switching processes is still missing. Within a good approximation of their formation process, nanofilament formation consists of multi-nanoparticle aggregation/assembly along a preferential axis, sometimes also referred to as coalescence. We adopt a classical molecular dynamics scheme, following a similar workflow as in coalescence and aggregation studies of nanoparticles, from the pioneering work by Mariscal *et al.*^31^ This model makes it possible to compare the assembly, which is the subsequent deposition of nanoparticles in a specific direction.

**Fig. 1:**
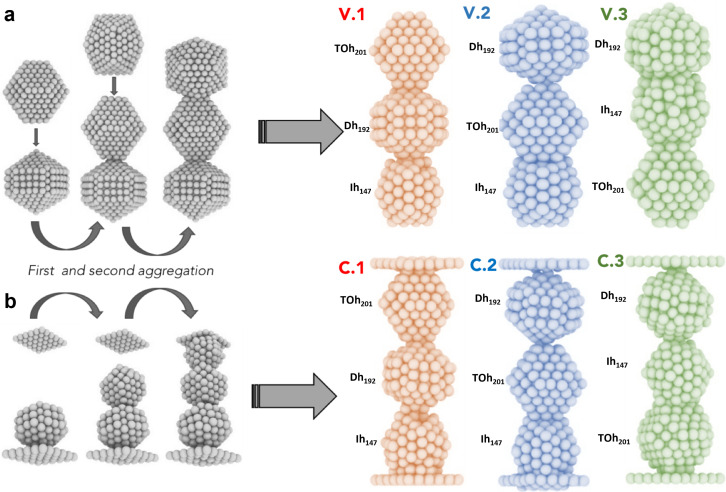
Assembly of individual Au nanoparticles in a vacuum and constrained between two (111) electrodes. (a) Free assemblies of the three initial shapes, Dh_192_, TOh_201_ and Ih_147_, are shown in a vacuum. The arrows represent the first and second aggregation along the preferential axis. The three initial configurations, labelled with V, are shown in the right panel. The colour identifies the ordering: green when Ih, blue when TOh, and red when Dh sit in the middle, respectively. (b) Constrained assembly between (111) electrodes of the three initial shapes, Dh_192_, TOh_201_ and Ih_147_, labelled with C. The colour code is the same as the vacuum case. In both cases, the assembly is performed at negligible kinetic energies so that there are no local reconstructions at the interfaces.

We employ molecular dynamics to monitor the morphological evolution of polycrystalline Au nanostructures used as building blocks of constrained Au filaments. Furthermore, molecular dynamics simulations reveal the structural dynamics of nanofilaments based on their individual components, as well as the conditions under which the break occurs. Structural integrity is at the origin of the conductivity of nanofilaments. Breaking/formation of nanofilaments destroys/creates conductive paths, hence tuning the resistive switching and dictating device performance.^26^ We compare when the assembly of individual nanoparticles occurs (i) in a vacuum without constraints (free coalescence), and (ii) under a geometric constraint, namely when the nanoparticles land between two fixed Au (111) layers displaced at a certain distance, *L*_fix_. We stress that the distance between (111) layers can only be changed by a few percentage points with respect to the nanofilament length, otherwise the nanofilament does not touch the layers and the assembly would not be constrained. Fig. 1a and b provide a pictorial description of the nanofilament aggregation in both scenarios. Whilst previous studies^32–35^ focused on coalescence, sintering, and assembly in a perfect vacuum, or nanoparticles deposited onto a surface,^36^ here we propose a numerical study where the assembly of individual nanoparticles occurs between two electrodes modelled as two (111) Au surfaces kept fixed during the simulation. Such a scenario aims at reproducing the last generation of nanodevices based on metallic networks.^12–14,16,20,37,38^ Specifically, our study aims at shedding light on nanoscale inter-cluster connections in Au-nanoparticles assembled in films below the percolation threshold.^12–14,16,20^ These films are granular and present nanoscale discontinuities, which make the system similar to a network of clusters bridged by nanofilaments. The nanofilaments bridging the clusters can be only a few nanometers long due to the percolating nature of these networks.^14^ These networks present a large number of interfaces, defects, and grain-boundary nano-junctions that resemble the assembly of clusters with different morphologies; hence, we decided to assemble filaments with highly symmetric, but different, nanoparticle morphologies. Other experimental settings^39–41^ investigate structures that are single-crystalline face-centred-cubic (FCC); hence, they do not consider possible FCC *versus* non-crystallographic interfaces, although they can occur in cluster-assembled films.

The main focus is to show how the order of the initial morphology of the seeds affects the structural evolution of the nanofilament. We study the assembly of Au nanoparticles with three different initial morphologies selected from among the most common geometries, namely icosahedra (Ih), decahedra (Dh) and face-centred-cubic (FCC)-like structures, such as truncated octahedra (TOh). We consider closed-shell shapes with 147 and 201 atoms with cross-sectional diameters (vertex-to-vertex) between 1.4–1.7 nm. The rationale behind our choice is two-fold. On one side, Ih, Dh and TOh are the most energetically favourable structural motifs. Moreover, they have been observed experimentally^42^ and are likely to be produced in several cluster sources and hence deposited. It is highly probable that such geometries are encountered during the assembly of nanoparticles into filaments and films.^12,43^ We observe that the relative order of how the initial seeds are assembled and a dependence on the distance between electrodes affect the overall structural stability of nanofilaments. There is a tendency for an assembled nanofilament to break when the morphological order of the individual building nanoparticles has a Dh in the middle, whilst a nanofilament with a central TOh and two (100) facets parallel to the (111) electrodes separating a Dh and Ih is more resilient to breaking. In the former initial configuration, two grains with different geometries are simultaneously formed. By changing the electrode distance between 55 and 56.5 Å in steps of 0.5 Å, we observe that at 56 Å there is a probability above 90% of breaking the assembled filament, compared to just 8% if the electrodes are at 55 Å. The dramatic increase in breaking probability is due to the low coordination of Ih/Dh interfaces and the higher average atomic strain in nanofilaments with differing lengths (55 to 56 Å) than the equilibrium length (≈54.2 Å or 56.5 Å), corresponding to 23 and 24 layers piled with respect to the (111) surface of the electrodes.

## 2 Methodology

We perform classical molecular dynamics of nanofilaments at 300 K, 600 K and 900 K in a time-frame of 100 ns. Nanofilaments are obtained *via* the assembly of three nanoparticles with different morphologies. In the following, we show results obtained at 600 K, the best temperature to analyse structural rearrangements in Au nanofilaments, since diffusion processes are boosted but we are still below Au’s melting temperature. We employ the classical molecular dynamics package available in LoDiS where the Au–Au interaction is modelled by a Gupta (or Rosato–Guillope–Legrand) potential, which is a semi-empirical many-body potential derived in the second-moment approximation of the tight binding model (SMATB).^44^ The values of the four parameters are taken from Baletto *et al.*^45^ as they are widely used and are able to reproduce diffusion properties. We set a time step of 5 fs and we employ the velocity-Verlet algorithm^46^ to calculate the atomic trajectories. The temperature is kept constant by an Andersen thermostat^47^ with a frequency of 10^11^ Hz, which does not alter the surface diffusion properties of adatoms.^48^ A pictorial description of the nanofilament assembly is given in Fig. 1a and b. In Fig. 1a, nanoparticles are assembled along a preferential axis one-by-one. In Fig. 1b, we repeat the process between two fixed (111) surfaces acting as electrodes. The two arrows on the left represent the aggregation steps in which this assembly is performed, whilst on the right we illustrate the resulting nanofilaments as assembled in a vacuum (V.1 TOh_201_–Dh_192_–Ih_147_, V.2 Dh_192_–TOh_201_–Ih_147_, and V.3 Dh_192_–Ih_147_–TOh_201_) and constrained (C.1 TOh_201_–Dh_192_–Ih_147_, C.2 Dh_192_–TOh_201_–Ih_147_, and C.3 Dh_192_–Ih_147_–TOh_201_). These acronyms will be used extensively in the next sections. During the assembly, the coalescence process is a soft process, meaning that the kinetic energy of the incoming nanoparticles is negligible. The time between two subsequent events is on the order of ps, in such a way that there are no local reconstructions of the first interface. We run four 100-ns-long independent simulations. We change the order in which the three shapes are assembled. In particular, we run 12 free (in a vacuum) and 36 constrained simulations. The latter comprise 12 simulations for each different distance between the electrodes, which is kept constant during the simulation. We choose *L*_fix_ to be 55 Å, 55.5 Å, 56 Å, and 56.5 Å. Considering the inter-layer spacing in FCC gold to be 
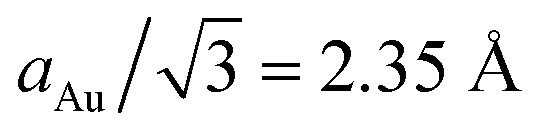
, the reference equilibrium lengths that can host an integer number of layers are 54.2 Å and 56.5 Å. Hence, the nanofilament strains calculated with respect to equilibrium lengths 54.2 Å and 56.5 Å are Δ*l*/*l* of 1.5%, 2.3%, 3.3%, and 0%. In this scenario, nanofilament breaking is energetically favourable as the number of surface atoms decreases and because smaller objects are more likely to rearrange over the electrode’s surface. The considered time scale (100 ns) is long enough to show significant differences between free and constrained scenarios. Our aim is to understand whether the formation of different interfaces, namely the nano-grain boundaries between TOh/Ih, TOh/Dh, and Dh/Ih, and with the electrodes (111)/Ih, (111)/Dh, or (111)/TOh, can alter the stability of the nanofilaments. We base our analysis on both energetic and geometrical descriptors. During the dynamics, the behaviour of the excess energy, *Δ*, is a good indicator of the relative stability of the individual nanoparticle and the assembled nanofilament. *Δ* is defined as^48^1
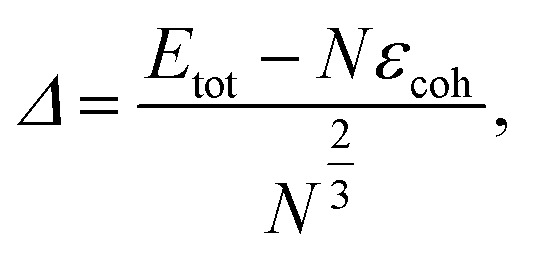
where *E*_tot_ is the total energy of the system and *ε*_coh_ is the bulk cohesive energy, one of the fitted parameters of the SMATB potential. A decrease in *Δ* indicates a favourable structural reordering. Due to the importance of discerning between amorphous and ordered shapes,^49,50^ we calculate the pair-distance distribution function (PDDF),2
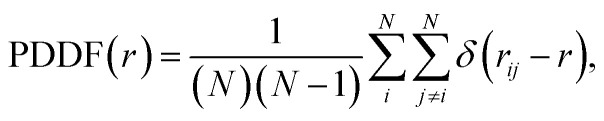
where *N* is the total number of atoms, and *r*_*ij*_ are the distances between *i* and *j* atoms. The binning is expressed as a Dirac delta-function, equal to 1 when *r* − *ε* ≤ *r* ≤ *r* + *ε*, with *ε* being the bin precision, which is set as 10^−2^ Å. In our recent work,^51^ we show that the absence of a peak at the bulk lattice value indicates the formation of a liquid-like state. We use the first minimum of the PDDFs to extrapolate the cut-off radius for the following Common Neighbour Analysis^52–54^ (CNA), employed to identify structural motifs. CNA provides insight on the local connectivity in a nanoparticle, providing each pair of nearest-neighbour atoms with a signature. The occurrence of different signatures enables the recognition of specific geometries.^55^ Precisely, the occurrence of (555) corresponds to local five-fold symmetry axes, which are typical of icosahedra, and are observed in decahedra to a lesser extent. (421) is typical of FCC motifs, whilst (422) represents grain boundaries, twinning planes and hexagonal close-packed (HPC) stacking faults. We describe the spatial extension of the assembled object through a deformation parameter,^56^3
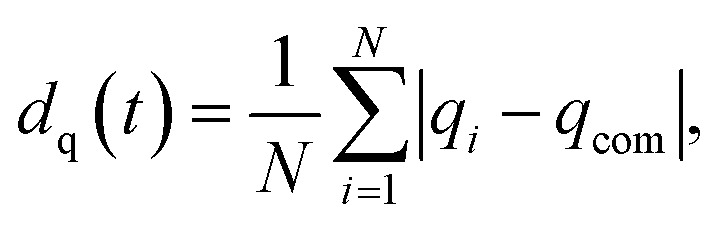
which quantifies the elongation of nanoparticles along each *q* = (*x*, *y*, *z*) direction, and by calculating the radius of gyration with respect to the centre of mass (com),4
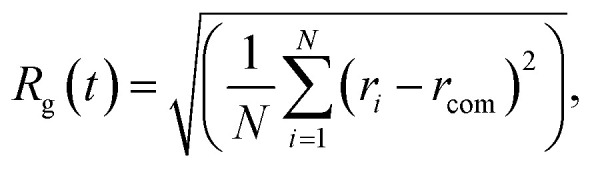
where (*r*_*i*_ − *r*_com_) is the instantaneous radial distance of the atom *i* from the centre of mass of the assembled system. For the constrained assembly, we perform a layer-by-layer (LBL) analysis. With the distance between the (111) electrodes being constant, we characterise the reordering processes sectioning the space between the electrodes in a series of layers parallel to them. The inter-layer distance along the *z*-direction for the slicing is 2.35 Å. We keep that value constant throughout the trajectory analysis. We record the number of atoms per layer and their coordination number^57–59^ per layer (CNL) with a cut-off length of 3.5 Å within the layer *l*:5
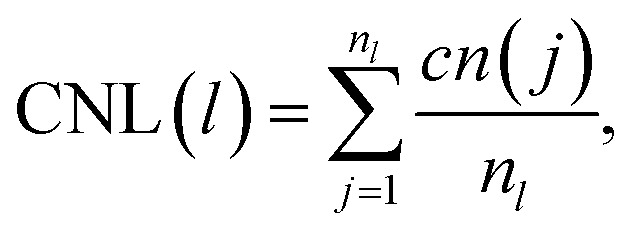
which is the sum of the nearest neighbours of each atom *j*, *cn*(*j*), in a layer *l* divided by the number of atoms in that layer (*n*_*l*_). The geometrically constrained dynamics is studied for different lengths. The nanofilaments have 6 × 6 Au (111) plates separated by (i) *L*_fix,1_ = 55 Å (1.5% strain), (ii) *L*_fix,2_ = 55.5 Å (2.3% strain), (iii) *L*_fix,3_ = 56 Å (3.3% strain) and (iv) *L*_fix,4_ 56.5 Å (0% strain). Au atoms in the (111) surfaces are kept fixed to mimic the presence of bulky electrodes. Hence, the surfaces do not participate in the dynamics. The purpose of using different distances is to monitor the effect of induced strain. In the ideal case, the expected number of layers between the two electrodes is, 
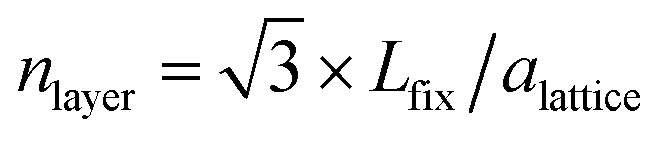
, where *a*_lattice_ is the bulk lattice parameter, which for Au is equal to 4.08 Å. For *L*_fix,1,2,3_, we expect to accommodate ∼23 layers parallel to the (111) plates, whilst in *L*_fix,4_, we can accommodate 24 layers. The largest induced strain is for *L*_fix,3_, as we have an elongation of 3.3% with respect to the ideal distance in bulk Au. If the nanofilaments do not break, we expect an average of 23 ± 1 atoms per layer, without considering atoms in the fixed (111) surfaces. Such characterization allows us to understand if the assembled nanoparticles adopt a (111) packing throughout the length of the filament, hence having a number of layers equal to the ideal number of layers and a FCC crystal coordination number of 12, besides trying to understand the role of Ih/Dh, Ih/TOh and Dh/TOh in nanofilament stability. For planes in between the electrodes, we expect a CNL close to 12 if the atoms pack according to the (111) electrodes. The (111) electrodes in turn should have coordination 9. We further analyze the nanofilaments in terms of atomic strain,6
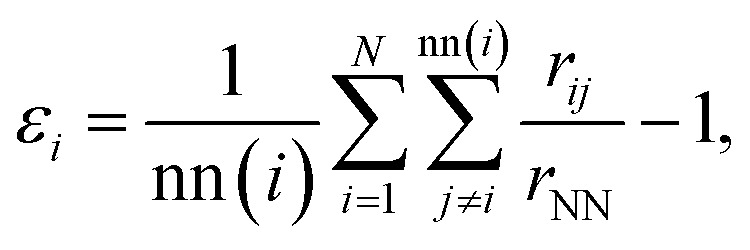
where the sum for atom *i* is over its nearest neighbours nn(*i*), and *r*_NN_ = 2.88 Å is the nearest-neighbour distance in bulk gold. This geometrical descriptor provides the strain at the atomic sites by comparing the calculated interatomic distance *r*_*ij*_ with the ideal distance in bulk gold; hence, large atomic strain values are expected in the area of the nanofilament that is going to break.

## 3 Results and discussion

**Fig. 2:**
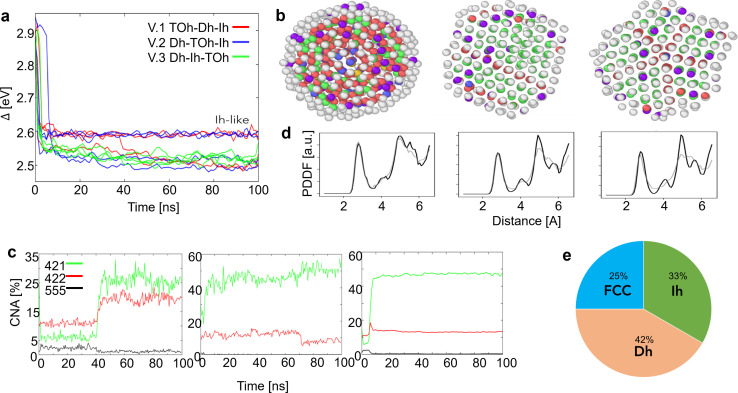
Assembly of three individual nanoparticles in a vacuum and their evolution at 600 K. (a) Excess energy *Δ* as a function of time. Colours indicate the initial ordering, as in Fig. 1. The plot highlights the efficiency of Ih_147_ to merge with both Dh_192_ and TOh_201_ simultaneously to form more energetically favourable shapes. (b) Structural analysis of V1, V2 and V3 (from left to right) with the PDDF (d) of both the initial (grey) and final (black) snapshots. (c) Common neighbour analysis *versus* time of three paradigmatic cases of V1, V2 and V3. They end in an icosahedral, FCC, and decahedral shape, respectively. The former cases (defected and incomplete Ih) is less energetically favourable and it occurs in 33% of the cases analysed (see pie-chart in (e)). Prototype final snapshots of V1, V2 and V3 are given with atoms classified in OVITO.^60^

We compare the gas-phase dynamics of free and constrained 540-atom-large nanofilaments assembled as depicted in Fig. 1. The study of larger nanofilaments can be found in Supplementary Information Section 2. We discuss the assembly of Dh_192_, Ih_147_, and TOh_201_ by changing the order with which individual morphologies are assembled during the formation process. Particularly, we consider cases with Ih_147_ (green), TOh_201_ (blue) and Dh_192_ (red) sandwiched in the middle, with the aim of investigating TOh/Dh, TOh/Ih and Ih/Dh interfaces and their role in nanofilament layering or breaking. Different nano-grain boundaries bear consequences for the stability of constrained nanofilaments.^61^ We maintain the same order of the nanoparticles between the free and the constrained cases to examine the evolution in time and to address if there is any peculiarity depending on the grain formed. Our simulations clearly show that in the absence of any constraint, the assembly of nanoparticles rearranges quickly from an elongated shape towards a spherical shape; see Fig. 2. The rearrangement occurs in less than 10 ns, as highlighted by the first major drop in the excess energy (Fig. 2a). Such a drop is evident in all cases, independently of the motif formed. We note that assembled nanoparticles that present an Ih-like shape are higher in energy, suggesting that on a longer time scale they might evolve towards Dh or FCC-like. Moreover, rearrangements and structural reconstructions can take place on a longer time scale of hundreds of ns or even longer, as recently pointed out by Ferrando *et al.*^35^ when studying the coalescence of two nanoparticles. Fig. 2b reports paradigmatic examples of Ih-, FCC- and Dh-like structures, together with the respective PDDF signatures (Fig. 2d). CNA signatures of the same structures are illustrated in Fig. 2c. We recall that 540 atoms is not a magic number for any of the most common morphologies (Ih, Dh, and FCC-like, such as octahedron or TOh). For the assembly of three nanoparticles with different initial morphologies and over a time scale of 100 ns, we do not notice a prevalent structural motif, as shown in Fig. 2e.

In all cases, the deformation parameter *d* along each Cartesian axis shortly converges to the same value, suggesting a shrink towards a spherical shape of about 2.5 nm (see Supplementary Information Fig. 12). This could be also quantified by the sharp decrease of the radius of gyration. This spherification process is accompanied by an increase in the occurrence of (421) and a decrease in (422) and (555) CNA signatures (Fig. 2c). The latter corresponds to the reduction or disappearance of local five-fold symmetries, and mainly icosahedral centres. The small percentage (∼1.5%) of the (555) indicates the existence of one five-fold axis, and hence the formation of defected decahedra. An absence of the (555) signature suggests the growth of FCC-like motifs, although combined with a significant amount of (422) which indicates grain boundaries. In all cases, the PDDF (Fig. 2d) shows that besides several defects that the assembly evolves among solid states, and also in the case of the formation of distorted Ih, there is a peak in the PDDF (Fig. 2d) corresponding to the Au bulk lattice. CNA analysis reveals different mechanisms of the reordering depending on the shape deposition, as well as differences in the final shape. Nonetheless, the time scale depends on the initial structural order, where a Dh in between seems to slow down the re-organisational process. Inter-cluster necks are quickly eliminated in the majority of the cases, depending on the geometries involved. Single and multi-fold twinnings are frequently detected, but in any event the icosahedral centre is lost, as highlighted by a decrease in the (555) CNA. We note that a tendency in reordering towards shapes with a large crystalline state is generally observed and quantified through an increase in the (421) CNA signature. The evolution of the excess energies with respect to time for three selected simulations, illustrated in Fig. 2a, indicates that coalescence considerably affects the formation of nanoparticles. This is due to atomic diffusion and the appearance of inter-cluster necks with a lifetime that depends on the type of surfaces in contact. If an icosahedral-like shape is not formed, the excess energy is higher (2.6 eV), against a value of ≤2.55 eV when Dh- or FCC-like motifs are formed.

If the assembly follows TOh_201_–Dh_192_–Ih_147_ (red lines in Fig. 2a), the nanoparticle formed after 100 ns is 25% decahedron, 25% FCC-like and 50% Ih-like. Generally, more time is needed to adjust into energetically more favourable structures. Nanofilaments with a decahedron placed in the middle also show a tendency to eliminate inter-cluster necks, except for the fact that reordering processes occur at a slower pace. In the case that Ih-like structures are not formed, the reordering mechanism is in two steps. First, a local re-ordering eliminates twinning-planes, reducing the overall (422) signature, and the second (slower) step almost eliminates any sign of (555) local environments. The latter is associated with a second drop in the excess energy around 40 ns. The structural outcome from Dh_192_–TOh_201_–Ih_147_ (blue lines Fig. 2a) is 50% decahedra and 50% defected Ih-like. Although not always taking place, the rearrangement is faster than when a Dh is in between. The disappearance of (555) occurs within 20 ns, if not less, at 600 K. The assembly following the Dh_192_–Ih_147_–TOh_201_ order (green lines Fig. 2a) leads to more symmetric structures. None show an icosahedral center after 100 ns. They display a finite number of hexagonal-close-packed (HCP) planes, sometimes parallel (FCC-like motifs) or five coinciding in one axis (Dh-like). Placing the icosahedron in the middle seems to ease the filling of the necks and the assembly process. Within 1 ns, solid-to-solid arrangements occur to destroy inter-neck grain boundaries and a quick atomic rearrangement leads to a Dh or a FCC-like morphology with equal probability. The rearrangement is as fast as 10 ns at 600 K. Summarising, we find that: (i) placing Ih_147_ in the middle hastens the coalescence with (100) and (111) facets of TOh_201_ and Dh_192_, respectively; (ii) other types of neck undergo morphological transformations at a lower pace and/or following more elementary steps. Each nanoparticle shows a solid phase (second peak of the PDDF corresponding to the bulk lattice value), and in all cases the (421) CNA signature increases with time. In agreement with other numerical studies, in a vacuum the coalescence tends to form spherical shapes with or without five-fold symmetry axes, on a short timescale (nanoseconds). We should notice that over a longer time-scale than the one considered here, the probability of assuming a FCC might increase.^35^

**Fig. 3:**
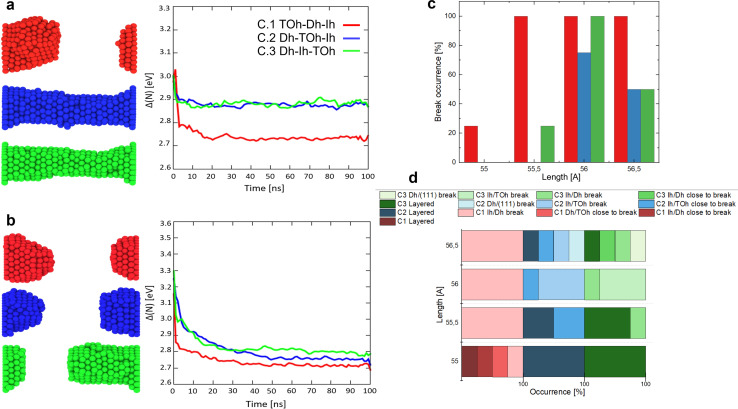
Paradigmatic snapshots after 100 ns and the evolution of the excess energy of C.1 (red), C.2 (blue), and C.3 (green) at the electrode distances (a) *L*_fix,1_ and (b) *L*_fix,4_. (c) Likelihood of breaking for various distances between the electrodes. Each bar is based on four independent simulations. (d) Percentage of broken, close to breaking and layered nanofilaments after 100 ns. The percentages are estimated independently of the electrode distance and of the initial morphological sequence. Overall, we observe better stability (layering) in C.2 Dh_192_–TOh_201_–Ih_147_ and C.3 Dh_192_–Ih_147_–TOh_201_.

On the other hand, the assembly of nanoparticles constrained between two electrodes does not evolve into spherical structures as in the free case, but rather shows a tendency to break the nanofilament or a layering process, in which atoms rearrange following the (111) orientation of the electrodes. Paradigmatic examples of the assembly of three nanoparticles between fixed gold plates are shown in Fig. 3a and b. The nanofilament break reduces the number of surface atoms and the surface energy contribution. Indeed, we see an energy drop associated with the breaking of nanofilaments in Fig. 3a and b. The energy threshold of Δ(*N*)_Th_ ∼ 2.8 eV identifies when the filament breaks, whilst nanofilaments with Δ(*N*)_Th_ > 2.8 eV show a layered structure. We find that the probability for a nanofilament to break depends both on the shape of each individual nanoparticle and the length of the nanofilament, as illustrated in Fig. 3c and d. The structures present holes, dislocations, and stacking faults (see Supplementary Information Section 4), which lead to the breaking of the nanofilament in 1/12 cases for *L*_fix,1_ = 55 Å and in 11/12 cases for *L*_fix,3_ = 56 Å. This corresponds to an 84% increase in break occurrence, which peaks at the length for which strain is maximum (*L*_fix,3_ = 56 Å, 3.3% from equilibrium distance). The relative order of the initial morphology in the filament strongly affects the probability of breaking it; see Fig. 3d. The breaking point can be predicted *via* the formation of less populated layers and a peak in the atomic tensile strain. If a layer contains <12 atoms ( hexagon with sides of less than 2 atoms), we classify it as “close-to-break”. We further classify nanofilaments as “layered”, “close to breaking” or “broken”. The color code for layered nanofilaments has darker intensities. Overall, 52% of nanofilaments break, whilst ∼10% are close to breaking and ∼38% undergo layering; see Fig. 3c and d. A TOh in the middle lowers the probability of breaking the nanofilament drastically, with no break occurrence at *L*_fix,1_ and *L*_fix,2_, 75% break occurrence at *L*_fix,3_ and 50% at *L*_fix,4_. In C.2, nanofilament breaks occur at the Ih/TOh interface or at the Dh/electrode interface. In C.1, the Dh in the middle is responsible for low-coordinated interfaces with both the Ih and the TOh, resulting in the nanofilament being more prone to breaking, with 25% break occurrence at *L*_fix,1_, and 100% at *L* > 55 Å. All break occurrences in C.1 are at the Ih/Dh interface. The nanofilaments with Ih in the middle show breaks at both Ih/Dh and Ih/TOh interfaces with no clear preference, showing that the most fragile interface is the one with Ih.

**Fig. 4:**
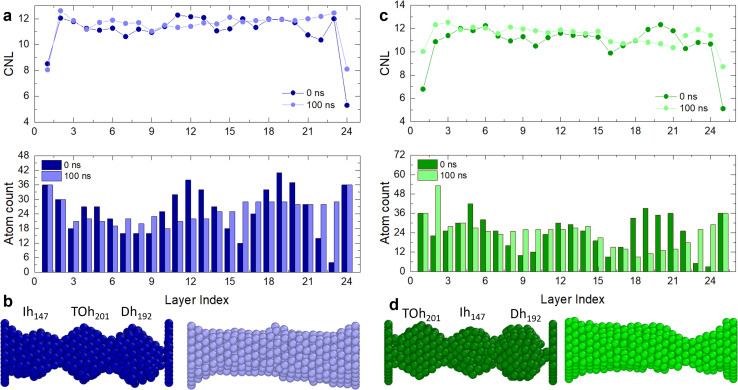
(a) CNL and atom count per layer of (b) C.2, which has a TOh_201_ in the middle, with length *L*_fixed,1_ = 55 Å. This is an example of layering in 24 layers with 26 ± 5 atoms per layer. (c) CNL and atom count per layer of (d) C.3, which has an Ih_147_ in the middle, with length *L*_fixed,4_ = 56.5 Å. This structure stratifies in 25 layers with 26 ± 11 atoms per layer and it is an example of a structure close to breaking, since layer 18 has <12 atoms and the CNL decreases to ∼10 between layer 18 and 21, at the Ih/Dh interface. The initial structures are dark, whilst lighter structures are after 100 ns.

**Fig. 5:**
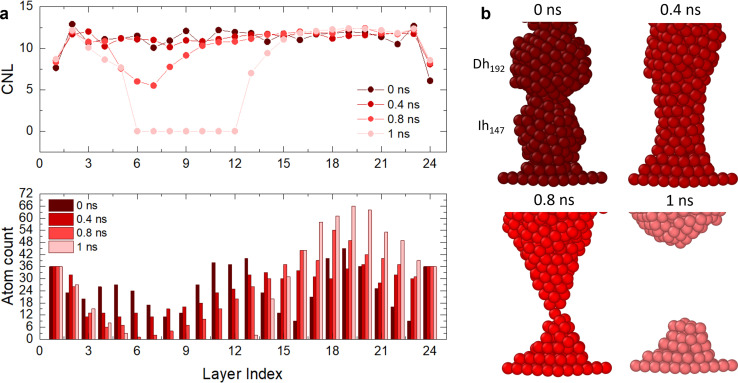
(a) CNL and atom count per layer of (b) C.1, which has a Dh_192_ in the middle, with length *L*_fixed,1_ = 55 Å. The nanofilament breaks within 1 ns at the Ih/Dh interface. The Ih/Dh interface progressively diminishes from 12 to 0 atoms, indicating atom migration towards the electrodes. The break is characterized by a decrease in CNL from 11 (0 ns), to 10.5 (0.4 ns), to 6 (0.8 ns), to 0 at layer 6.

To shed light on the layering and break dynamics, we count the number of atoms per layer and the coordination number per layer to understand the inter-layer diffusion mechanisms, particularly at the interfaces. For perfectly layered FCC structures without any vacancies, we expect ∼23 layers for *L*_fix,1_ = 55 Å, *L*_fix,2_ = 55.5 Å and *L*_fix,3_ = 56 Å, and ∼24 layers for *L*_fix,4_ = 56.5 Å. Since we have 612 atoms, we expect ∼26 atoms per layer, which becomes ∼23 if we subtract the number of atoms in the electrodes. However, in the ideal case where atoms rearrange as (111) planes, these layers should contain hexagons with sides between 3 atoms (19 atoms, 12 at the surface and 7 in the bulk) and 4 atoms (38 atoms, 19 at the surface and 19 in the bulk), leading to ∼55% of atoms in the nanofilament being at the surface. A large number of atoms at the surface means high surface tension; hence, the interplay of the different interfaces composing the nanofilaments plays a key role in the evolution of the structure, including the number of layers, the number of atoms per layer, their coordination and consequently the breaking or layering of the nanofilament. Fig. 4 and 5 show paradigmatic examples of a layered nanofilament (C.2, blue), a nanofilament close to break (C.3, green) and a sub-ns break (C.1, red). We observe the formation of 24 layers for *L*_fix,1_, *L*_fix,2_ and *L*_fix,3_ and 25 layers for *L*_fix,4_, which is one extra layer compared to the ideal case. We attribute this to the presence of vacancies and stacking faults in the nanofilament, which prevent layers from stacking in a close-packed structure. The presence of stacking faults changes the spacing between layers, which results in a deviation from the ideal number of layers. Indeed, we observe ∼26 atoms per layer, which is far from the number of atoms in hexagons with sides of 3 or 4 atoms expected in ideal (111) layers, indicating the presence of vacancies and dislocations. The electrodes layers, namely layer 1 and 24, have a fixed number of 36 atoms and form a perfect (111) plate.

For C.2 (Fig. 4a and b, blue case), the electrode CNL ∼ 8 indicates that the layer next to it is not piling with respect to a (111) arrangement, as otherwise a CNL of 9 would be observed. This suggests that electrode layers are coordinated to their neighbour layers as cubes or hexagonal bipyramids, as the next layer tries to mimic the electrode but contains fewer atoms. At the beginning of the simulation, the Dh/(111) interface (layer 24) has CNL ∼ 6, as the pyramid vertex of Dh_192_ is attached to the electrode, meaning that the nearest neighbours are intra-layer. Overall, we observe an increase in CNL in layers that start with low coordination, namely the Dh/(111), Ih/TOh and TOh/Dh interfaces. 16 layers have CNL ∼ 12, while 6 have CNL ∼ 11. In contrast, we observe a decrease in the CNL (or no change) in the layers within the TOh, the Dh and partially the Ih (layers 3 and 4). This suggests that atoms migrate to the interfaces, creating vacancies in highly coordinated sites in the attempt to form a (111) order. In C.3 (Fig. 4c and d, green case), the (111)/TOh interface has CNL = 10, whilst the (111)/Dh interface has CNL = 9. Similarly to C.2, the CNL increases in layers with low coordination (CNL < 12) or remains constant for layers with CNL = 12. This occurs at the TOh/Ih interface. However, there is a decrease in CNL (<11) at the Ih/Dh interface and the layers corresponding to the Dh. This indicates a migration from the Dh to the TOh/Ih interface, which grows and achieves CNL ∼ 12. We classify this nanofilament as close to breaking due to the decrease in CNL and the low atom count (<12) in the proximity of the Ih/Dh interface. In C.1 (Fig. 5), the nanofilament breaks in <1 ns at the Dh/Ih interface. The break is anticipated from a decrease in CNL for the layers in the proximity of the Dh/Ih interface. At 0 ns, the Dh/Ih interface (layers 6–7) have CNL ∼ 11. The CNL of these layers progressively decreases to ∼6 at 0.8 ns. A CNL of ∼8 is then observed for the layers formed at the surface after the break, indicating 4-fold symmetry and a (100) arrangement. In contrast, layers close to the (111) electrodes have CNL ∼ 12, indicating a FCC arrangement. The electrodes reach CNL ∼ 9. This confirms that the nanofilament arranges in a close-packed structure near the electrodes, but the low coordination between Dh and Ih due to the high number of vertices (12 for Ih and 10 for Dh) together with the non-ideal number of atoms in the nanofilament is such that the nanofilament breaks, leading to more atoms on the surface, minimizing its excess energy, and continuing the (111) layering and clustering over the electrodes.

**Fig. 6:**
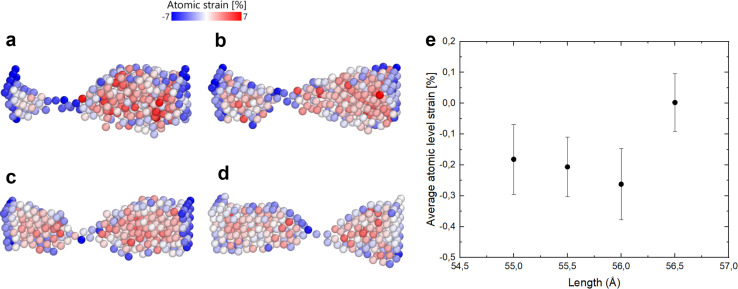
Atomic strain calculation of (a) C.1, *L* = 55 Å, (b) C.1, *L* = 55.5 Å, (c) C.2, *L* = 56 Å, and (d) C.2, *L* = 56.5 Å on a scale from −7% to 7%. Strain is not equally distributed along the nanofilament, with the largest values of tensile strain at the breaking interface and along the surface. The snapshots are sliced in OVITO by 1.2 Å to display bulk atoms. (e) Average atomic strain for each length within 750 ps before nanofilament breaking.

Indeed, if the nanofilament is longer, we expect more atomic migration towards the (111) plates, which will increase the probability of breaking. For the TOh–Dh–Ih nanofilament (C.1), the reason why we observe a rise in the probability of breaking is due to the Ih/Dh interface, which is already low-coordinated at the start of the simulation due to the small structural change. For this type of nanofilament, we observe a break at the Ih/Dh interface after 25 ps for the longer filaments. Atoms in the Ih migrate towards the closer electrode, whilst the Dh coalesces with the TOh. For the other nanofilaments, the rise in the probability of breaking is driven by the increased strain at the atomic level (eqn (6)). The small 0.5 Å structural change causes the interatomic distances between nearest neighbours to be different from the equilibrium distance (2.88 Å). Fig. 6 shows four paradigmatic snapshots of nanofilament breaking for (a) C.1, *L* = 55 Å, (b) C.1, *L* = 55.5 Å, (c) C.2, *L* = 56 Å, and (d) C.2, *L* = 56.5 Å, together with the average atomic strain for each length. We observe that the actual strain at specific atomic sites can be larger than the strain calculated as 
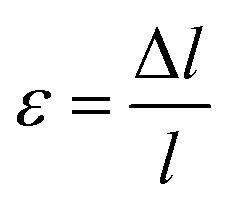
. Specifically, we found that the atomic strain at the breaking junction is ≈−7% for all nanofilaments that break. This is much larger than the strain calculated as 
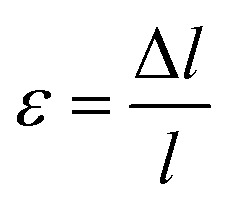
. Additionally, nanofilaments with lengths different from the equilibrium length (55 Å to 56 Å) are subject to more average atomic strain the greater the distance from the equilibrium length. Specifically, we calculated −0.18%, −0.21% and −0.26% for 55 Å, 55.5 Å and 56 Å respectively. In line with our data and interpretation, there are several papers that support the idea of uneven strain distribution and breaking due to atomic surface diffusion driven by energy minimization. Molecular dynamics simulations by Wang *et al.*^17^ show that ps-scale nanofilaments break at *T* = 800 K, which they attribute to clustering effects and atomic surface diffusion. Palmer *et al.*^62^ also simulated the breakage of metallic nanofilaments in the nanosecond range, but they focused on highly symmetric FCC filaments only. A combination of TEM and atomistic simulations demonstrated that uneven tensile strain triggers twin boundary migration and dislocation slipping at the atomic scale,^63^ specifically in penta-twinned nanoparticles. Self-assembled nanofilaments have a multitude of atomic twin boundaries with a 5-fold symmetry, shown by our CNA analysis for simulations without geometrical constraints. There is an indication that 5-fold symmetries, which belong to decahedra and icosahedra, are responsible for an uneven distribution of tensile strain in self-assembled nanofilaments.^63^

Saying that, small variations in the length of the nanofilament can increase the likelihood of breaking considerably, especially when decahedron junctions are present. For 56.5 Å long filaments, strain is not the driving force of breaking, but the increased surface length is such that breaking is extremely favourable for energy minimization. However, in the latter case the probability of breaking is still less than that of the 56 Å case. Increasing the size of the individual nanoparticles will likely reduce the possibility of breaking due to the smaller surface-to-volume ratio of larger objects. However, when Dh nanoparticles are involved in the assembly, even in filaments with larger size, we expect a larger probability of breaking compared to filaments with FCC or Ih only. This is because it is known that diffusion is faster along grain boundaries;^64^ hence, the larger Dh, which possess twinned planes, would likely cause an increased probability of breaking in larger filaments and networks. Since structural integrity is at the origin of Au network conductivity, and structural integrity is affected by nanoparticle morphologies, our results are useful for designing devices with resistive switching behaviour. We propose the following design of experiments. Nanoparticle films well below the percolation threshold are deposited over an array of inter-digitated electrodes. Many nanoparticle–electrodes contacts would be present and can be characterized electrically. This method, while it does not guarantee that exact Ih–Dh–TOh nanoparticle filaments will be measured, will certainly allow exploration of the effects of these shapes and their possible breaking on electronic transport. The exact assembly of Ih–Dh–TOh filaments on top of electrodes can potentially be achieved by employing a transfer technique based on a PDMS stamp and a colloidal solution of nanoparticles. This approach has already been used to transfer single 60 nm Au nanoparticles on top of photonic structures.^65^

## 4 Conclusions

Our work highlights a fundamental difference between the coalescence of Au nanoparticles with and without geometrical constraints, which extends to all nano-assembled metallic systems. With no geometrical constraints, we observe a spherification process characterized by an increase in the FCC local environment and a decrease in dislocations and 5-fold symmetries, as quantified by the evolution of (421), (422) and (555) CNA signatures over time. When the Au-nanoparticle assembly occurs between (111) electrodes, the formed filament will arrange in a FCC (111) arrangement, or break into two subsystems wetting the electrodes. Nano grain boundaries formed by different morphologies and strains cannot be neglected, as they affect the timescale and probability of breaking. We found that Dh/Ih interfaces are more likely to break than Ih/TOh and Dh/TOh interfaces and that strain causes an 82% increase in breaking probability. The small strain we apply by constraining the nanofilament to fit within a certain distance causes an increase of the average atomic strain, which reaches −7% at the breaking junctions. Our atomistic description provides important insights on breaking/formation mechanisms of nanoparticle junctions assembled into nanofilaments and larger networks, which may find application in metal-only non-volatile memory devices and neuromorphic devices. Our observations suggest that the breaking of Au-nanoparticle-assembled filaments is a likely event at finite temperatures due to surface diffusion, and the interplay of low-coordinated junctions and interatomic strain (*i.e.*, the imposed length of the filament between electrodes). This can drive the electrical behaviour of metallic-nanoparticle-assembled networks, in particular resistive switching due to formation/breaking of conductive paths.

## Data availability

Supplementary information for this article is available at: https://zenodo.org/doi/10.5281/zenodo.12772974.

LoDiS, Low dimensional systems molecular dynamics code: Developed by Francesca Baletto’s groups at King’s College London until August 2021 and after that at Univ. of Milan. Developers and main users: K. Rossi, L. Pavan, R. M. Jones, M. Tiberi, S. Zinzani, R. Pinto-Miles, *etc.* Available at https://github.com/kcl-tscm/LoDiS.

Sapphire: Developed by Francesca Baletto’s groups at King’s College London until August 2021 and after that at Univ. of Milan. Main developers: R. M. Jones, C. Zeni and M. Tiberi, and for a newer version, S. Zinzani. Available at https://github.com/kcl-tscm/Sapphire.^50^

## Conflicts of interest

The authors have no conflicts to declare.
